# Nicotine exposure during differentiation causes inhibition of N-myc expression

**DOI:** 10.1186/1465-9921-14-119

**Published:** 2013-11-05

**Authors:** Ahmi Ben-Yehudah, Becki M Campanaro, Laura M Wakefield, Tia N Kinney, Jill Brekosky, Vonya M Eisinger, Carlos A Castro, Diane L Carlisle

**Affiliations:** 1 Division of Developmental and Regenerative Medicine, Department of Obstetrics, Gynecology, and Reproductive Sciences, University of Pittsburgh, Pittsburgh, PA 15213, USA; 2 Pittsburgh Development Center, Magee Womens Research Institute and Foundation, Pittsburgh, PA 15213, USA; 3 Department of Neurological Surgery, University of Pittsburgh, A505 Scaife Hall, 1550 Terrace Street, Pittsburgh, PA 15213, USA

**Keywords:** Embryonic stem cells, Directed differentiation, Lung development, Nicotine, Cell cycle

## Abstract

**Background:**

The ability of chemicals to disrupt neonatal development can be studied using embryonic stem cells (ESC). One such chemical is nicotine. Prenatal nicotine exposure is known to affect postnatal lung function, although the mechanisms by which it has this effect are not clear. Since fibroblasts are a critical component of the developing lung, providing structure and secreting paracrine factors that are essential to epithelialization, this study focuses on the differentiation of ESC into fibroblasts using a directed differentiation protocol.

**Methods:**

Fibroblasts obtained from non-human primate ESC (nhpESC) differentiation were analyzed by immunohistochemistry, immunostaining, Affymetrix gene expression array, qPCR, and immunoblotting.

**Results:**

Results of these analyses demonstrated that although nhpESCs differentiate into fibroblasts in the presence of nicotine and appear normal by some measures, including H&E and SMA staining, they have an altered gene expression profile. Network analysis of expression changes demonstrated an over-representation of cell-cycle related genes with downregulation of N-myc as a central regulator in the pathway. Further investigation demonstrated that cells differentiated in the presence of nicotine had decreased N-myc mRNA and protein expression and longer doubling times, a biological effect consistent with downregulation of N-myc.

**Conclusions:**

This study is the first to use primate ESC to demonstrate that nicotine can affect cellular differentiation from pluripotency into fibroblasts, and in particular, mediate N-myc expression in differentiating ESCs. Given the crucial role of fibroblasts throughout the body, this has important implications for the effect of cigarette smoke exposure on human development not only in the lung, but in organogenesis in general.

## Background

Maternal exposure to nicotine is known to lead to alterations in postnatal lung function. Nicotine freely passes through the placenta and can accumulate in amniotic fluid [1,2]. Studies using non-human primates have shown that nicotine exposure during development leads to the overexpression of some nicotinic acetylcholine receptors (nAChR) as well as some specific genes and proteins such as collagens in the developed lung [3-5]. In addition, pulmonary function assessments of non-human primates exposed to prenatal nicotine demonstrated decreased forced expiratory flows that mimic the decreased forced expiratory flows measured in human infants who were exposed to maternal cigarette smoke [3], implying that among the many chemical present in cigarette smoke with the potential to affect development, nicotine may have a significant role in the decreased pulmonary function in infants exposed to maternal smoking. However, the mechanisms by which nicotine affects lung development are not well understood.

The ability to use embryonic stem cells (ESCs) as tools for the study of developmental biology and, in particular, as sensors to determine the adverse effects of chemical exposures during development was recognized even before primate embryonic stem cells were first derived [6]. ESCs are now in use for the study of developmental cardiotoxicity and neurotoxicity [7-10], since studies of the earliest stages of embryonic development and the effect of environmental exposures are difficult in animals and impossible in humans. Directed-differentiation protocols shed insight into these early stages by allowing investigation of differentiation of ESCs as they move towards more mature cell types ([11] for review). As protocols for differentiating ESCs become more established, we can move our focus to using cells in these models to examine the effects of chemical exposures during the differentiation process. In this study, we focus on the differentiation of ESC into fibroblasts. Fibroblasts are a critical component of the lung and direct epithelialization of the lung through paracrine and other methods, and prenatal nicotine exposure has been demonstrated to increase collagen and airway wall thickness, affecting airway resistance [4]. Thus, changes in fibroblast phenotype during development may affect lung function after birth.

Non-human primate embryonic stem cells (nhpESC) are available [12-21] and provide an opportunity to correlate *in vivo* physiologic data from previous studies with analysis of intracellular signaling. We have published a series of related nhpESC lines derived from *Rhesus macaca*[12,22]. These lines are more than 95% identical in their gene expression patterns, making them ideal for array analysis of gene expression [22,23]. In this study we use these lines as sensors to assess alterations in differentiation that result from nicotine exposure.

NAChR have been shown to be expressed on many cell types, and the endogenous ligand, acetylcholine, is expressed ubiquitously (reviews by [24-30]). Nicotine exposure *in vitro* leads to alterations in the differentiation of keratinocytes [31,32] and causes release of chromogranin A from pheochromocytoma cells [33]. Thus, nicotine has been previously demonstrated to affect cellular differentiation processes in some somatic tissues, although neither nhpESC nor hESC have been examined for the expression of nAChR or for the effect of nicotine on the differentiation process. In this study, the expression of nAChR was confirmed in nhpESC, and directed differentiation of nhpESC into fibroblasts was carried out in the presence or absence of nicotine. Microarray analysis of the resulting differentiated cells includes a number of previously unreported targets of nicotine regulation. These changes are specific to the differentiation process, and alterations of these genes by nicotine during development could contribute to the decreased lung function seen in infants exposed to maternal nicotine.

## Methods

All experiments involving animals were approved by the Institutional Animal Care and Use Committees (IACUCs) from the Magee-Womens Research Institute and the University of Pittsburgh and experiments involving embryonic stem cells were approved by the University of Pittsburgh Human Stem Cell Research Oversight (hSCRO) committee.

### Cell lines

NhpESC cell lines were a generous gift from Gerald Schatten (U. Pittsburgh). NhpESC lines were previously described ([12], personal communication). Primary adult non-human primate lung fibroblasts were obtained during necropsy by taking tissue biopsies. Tissues were minced and grown in DMEM with 15% serum, 1% penicillin/streptomycin, and 2 mM L-glutamine.

### NhpESC culture

NhpESCs were cultured in 80% KO-DMEM (Invitrogen, Carlsbad, CA), 20% Knockout Serum Replacement (Invitrogen), 1 mM L-glutamine (Invitrogen), 0.1 mM non-essential amino acids (Invitrogen), and 4 ng/ml basic human recombinant FGF (Invitrogen). NhpESCs were grown on inactivated mouse embryonic feeder cells (MEF’s) and manually passaged weekly. Media was replaced every 24 hours [12].

### Pluripotency marker expression

NhpESCs were fully characterized to demonstrate pluripotency using a teratoma assay [22]. To ensure nhpESCs maintained a pluripotent phenotype throughout the experiments, cells were stained for the positive pluripotency markers: Oct-4, SSEA-4, Tra 1–80 and Tra 1–61, as well as the negative nhpESC marker SSEA-1. Immunocytochemistry was performed as follows: Culture dishes containing undifferentiated colonies were fixed by addition of either 100% methanol at -20°C for 15 min, or 2% paraformaldehyde in PBS for 40 minutes followed by a 15 min wash in PBS + 1% Triton X-100 (PBS-Tx, Sigma, St. Louis MO). After fixation, non-specific binding of the primary and secondary antibodies was blocked by 30 minutes incubation in PBS with 0.5% goat serum. Primary antibodies were diluted in PBS-Tx and incubated on the coverslips for 40 min at 37°C in a humidified chamber, with the exception of Oct-4 which was incubated overnight at 4°C. Primary antibodies were detected with fluorescently labeled appropriate secondary antibodies. DNA was detected with 5 μM TOTO-3 (Molecular Probes, Eugene OR). Coverslips were inverted onto slides and mounted in Vectashield anti-fade medium (Vector Labs, Burlingame, NH) to prevent photobleaching [22].

### *In Vitro* differentiation

Colonies were manually passaged and dissociated into small clusters. They were cultured in non-adherent dishes to form embryoid bodies in differentiation medium (80% KO-DMEM, 1 mM L-glutamine, 20% FBS, 1% non-essential amino acids). After 4 days in suspension, aggregates were transferred onto gelatin coated culture dishes and cultured for an additional 9 days in differentiation medium. Outgrowth cultures were manually passaged by scraping cells with a differentiated phenotype from the periphery of the colonies, and these differentiated cells were placed in a new culture plate in differentiation medium. After the first passage, cells were grown in standard fibroblast media (90% KO-DMEM, 10% FBS, 1% non-essential amino acids, 2 mM L-glutamine) and passaged at 80% confluency using trypsin. Cells were banked at each passage and were cultured until they stopped growing or through passage 10. Experimental groups were treated with 100 nM nicotine starting on day 1 of the differentiation protocol.

### PCR for nAChR

RT-PCR for nAChR was done using primer sequences that have been previously published [34]. All primers span introns and do not amplify DNA. GAPDH or actin was always used as a positive control for RNA integrity. Oligo dT12-18 (Invitrogen) was annealed to 1 μg total RNA and reverse transcribed with Superscript II (Invitrogen). The reaction contained 1 μg RNA, 500 ng Oligo dT12-18, 50 mM Tris–HCl, pH 8.3, 75 mM KCl, 3 mM MgCl2, 10 mM DTT, 1 mM each dNTP, 200 U superscript. Briefly, total RNA was incubated with oligo dT12-18 at 70.0°C for 10 min. The cDNA produced was then used as a template for PCR using specific primers. PCR amplification was performed in a 20 μl reaction containing 2 μl of the RT reaction, Taq DNA polymerase (Perkin Elmer), 1X PCR buffer, 1.5 mM MgCl2, 1 mM each dNTP and 1 μM primer. PCR was carried out in a Perkin-Elmer 9700 Thermocycler with 2 min, 95.0°C denaturation, followed by 30 cycles of 94.0°C for 30 s, 55.0-62.0°C (see [34]) for 30 s and 72.0°C for 30 s. Final extension was at 72.0°C for 5 min. 10 μl of each reaction was run on a 1% TBE gel for analysis. β2 and δ subunits were detected using nested PCR. Primary PCR reactions were carried out as described above. 2 μl of the primary reaction was used as the template for the secondary PCR reaction/second round PCR. Thirty rounds of PCR were carried out.

### Teratoma analysis

Differentiated fibroblasts were tested for their ability to form teratomas in the testes of mice. The analysis was done at the MWRIF Transgenic Core Facility according to their standard methods [12,22,35-38]. Pathology on the testes was performed by the MWRIF Histology Research Core Facility.

### Gene expression analysis

Gene expression analysis was performed by the DNA Analysis group at the University of Pittsburgh Genomics and Proteomic Core Laboratory using cell pellets provided by D. Carlisle’s laboratory as described herein. RNA was isolated from *in vitro* differentiated fibroblasts from three different nhpESC lines, each differentiated in the presence and absence of nicotine: nhpESC2706, nhpESC3106 and nhpESC4706. Three technical replicates were done with each cell line and condition, and cultures with and without nicotine were matched for passage number after differentiation. RNA Isolation: RNA was purified using a modified Trizol extraction method (Invitrogen, Carlsbad, CA). Briefly, suspended cells were extracted in 1 mL of Trizol with the addition of 200 μg of GlycoBlue (Ambion Inc, Austin, TX) added to each sample as a nucleotide carrier. After aqueous phase separation, the samples were incubated overnight at 20°C in 500 μl of isopropanol (100%) to precipitate the RNA. The RNA was then pelleted by centrifugation (14,000 g × 15 min), washed in 1 mL of 75% ethanol, and resuspended in 20 μl nuclease-free water at 45°C for 5 minutes. The RNA concentration and quality was evaluated with criteria for inclusion in subsequent *in vitro* transcription (IVT) assays comprising spectrophotometric absorption ratio of 260/280 >1.8 (NanoDrop, Wilmington, DE) and a RIN value of >8.0 via electrophoretic analysis (Agilent Bioanalyzer 2100, Agilent Technologies, Santa Clara, CA). Affymetrix Eukaryotic Target Preparation and Hybridization: *In vitro* transcription was performed using the Ambion Message Amp II-Biotin Enhanced Assay protocol starting with 100 ng of purified total RNA. Confirmation of cRNA diversity was obtained using the Bioanalyzer 2100 to generate an electrophoretogram for each IVT reaction regarding sample yield, integrity, and size diversity against a Universal Human Reference RNA (Stratagene, La Jolla, CA). Fifteen micrograms of purified, biotin labeled cRNA was fragmented and hybridized on Rhesus Macaque Whole Genome Arrays (Affymetrix Corp., Santa Clara, CA) for 18 hours. Washing, staining and scanning of arrays were performed on the Fluidics Station 450 and Scanner 3000 immediately after completion of hybridization. Microarray data was processed using GeneChip Operating Software (GCOS, Affymetrix) with signal intensity calculated by Microarray Suite version 5.0 (MAS 5.0).

### Statistical analysis

Differential gene expression analysis was performed in consultation with the University of Pittsburgh Genomics Analysis Services using BRB Array Tools from NCI (http://linus.nci.nih.gov/BRB-ArrayTools.html), and genes were selected at p < 0.001 (Additional file 1: Table S1). Statistical analysis of qPCR and immunoblotting was done by t-test, and analysis of slope for doubling time was done using a two-factor ANOVA for interaction on log-transformed cell counts.

### Quantitative PCR

Cells were pelleted by centrifugation at 200 × g for 5 min. RNA was isolated using Trizol (Invitrogen). Briefly, 1 ml of Trizol was added per cell culture dish and vortexed to lyse cells to homogeneity. 200 μl of chloroform (Sigma) was added followed by mixing and centrifugation for five min at 25,000 × g. The RNA containing supernatant was removed and the RNA pelleted using 600 μl of 100% isopropanol added to the supernatant and incubated at -20°C for at least 4 hrs. The sample was centrifuged twice at 13000 × g for 30 min at 4°C, with an ethanol wash in between, followed by air-drying. The RNA pellet was reconstituted in nuclease free water and treated with 1 μl DNase I for 30 min at 37°C. cDNA was prepared using the Improm-II Reverse Transcription System (Promega, Madison WI), according to manufacturer’s directions. Validated primer sets were purchased from Applied Biosystems and were used on an ABI 7900HT real-time PCR machine. Real-time data was analyzed and fold change was calculated using the comparative C_T_ method with either beta-actin or GADPH as the internal control [39].

### Protein isolation and immunoblotting

Proliferating cells at passage 8, 9, or 13 were scraped from flasks in ice cold PBS/Phosphatase Inhibitors. Cell pellets were lysed in RIPA Buffer supplemented with a Protease Inhibitor Cocktail (Roche). The lysate was centrifuged for 20 minutes at 14,000 × g and 4°C in a precooled microcentrifuge. Protein concentrations of the clarified lysates were quantitated using the BCA Assay Kit (Thermo Scientific) and a BSA standard curve following the manufacturer’s instructions. Protein samples were stored at -80°C. Samples for Western blot analysis were diluted in 4 × Laemmli buffer (BioRad) supplemented with 10% β-mercaptoethanol to a concentration of 10 μg of protein in 20 μL. Samples were heated at 55°C for 5 minutes and were separated on 10% polyacrylamide gels by SDS-PAGE and transferred to Immobilon-FL PVDF membranes (Millipore) by BioRad wet transfer. The membranes were blocked with LiCor Odyssey blocking buffer for 1 hour at room temperature and were then probed with a primary N-myc antibody (NCM II 100, Millipore) diluted 1:100 in LiCor Odyssey blocking buffer with 0.05% Tween. Membranes were washed three times for 15 minutes each in PBS supplemented with 0.05% Tween (TBST) and were subsequently probed with goat anti-mouse IR-Dye 800cw labeled secondary antibody diluted 1:30000 in LiCor Odyssey blocking buffer. Washes were repeated after labeling with secondary antibody three times in PBST and then once in PBS. Membranes were imaged using LiCor scanner. Membranes were stripped using LiCor NewBlot stripping buffer for 15 minutes at room temperature. Membranes were reprobed with mouse anti-β-actin diluted 1:10000 in LiCor Odyssey blocking buffer with 0.05% Tween followed by washes and probing with the LiCor secondary antibody, then washed and imaged as described above.

### Analysis of culture doubling time

For growth analysis, cells were plated in triplicate in 6-well plates (Corning Life Sciences, Corning, NY) at a concentration of 2 × 10^5^ cells/well. After 2, 3, 4, 5, 6, and 7 days, cells were trypsinized and counted using the Countess Automated Cell Counter (Invitrogen). Cell counts were analyzed by logistic regression and doubling times were calculated using http://www.doubling-time.com/compute.php.

## Results

### Nicotine receptors are expressed on stem cells before and after differentiation

**Figure 1:**
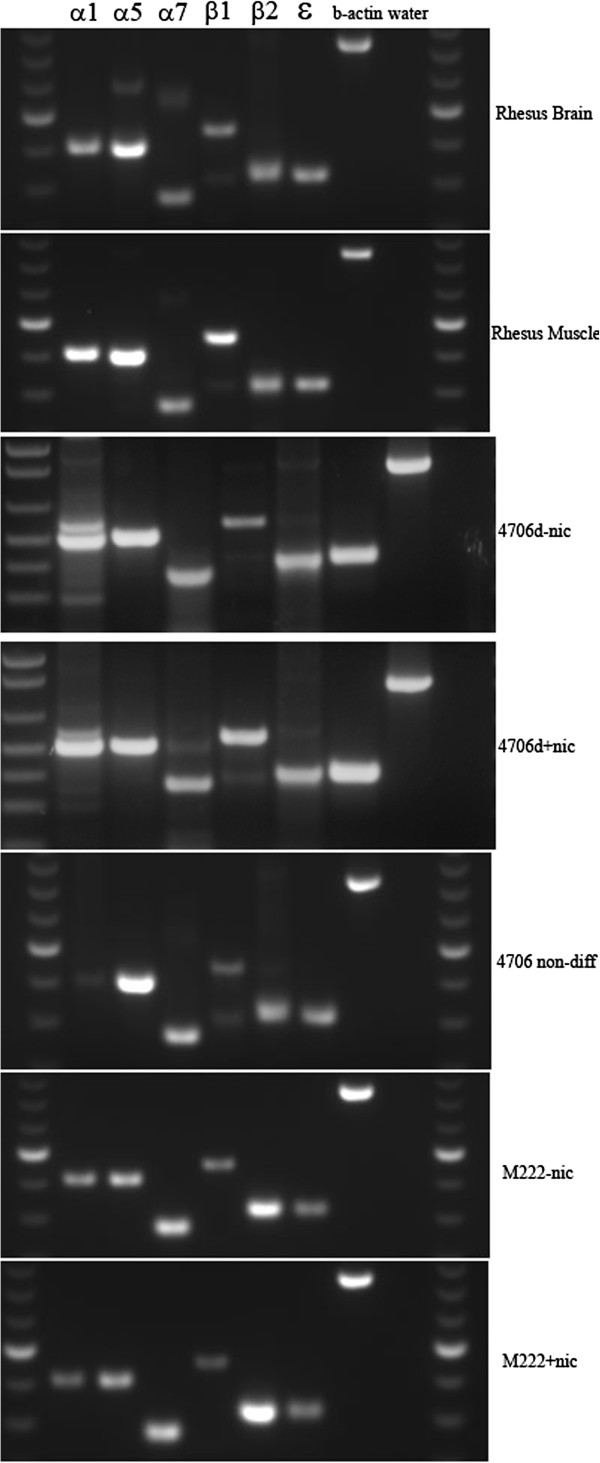
**nhpESC express nAChR.** We analyzed nAChR receptor subunit expression by RT-PCR. Brain and muscle serve as positive controls. Bands were sequenced and demonstrated that the correct gene was amplified. B-actin was used as a control for RNA quality, and water-only reaction was the negative control.

Lung fibroblasts are known to express nAChR as are mouse embryonic lung cells [34,40]. The most commonly expressed receptors in lung fibroblasts are those that make up the muscle type nAChR and the alpha7 neuronal nAChR [34,40]. Primate embryonic stem cells, including human and non-human primate, have not previously been examined for expression of nAChR. Based on our previously published results [34], we performed RT-PCR on the six subunits most likely to be found in fibroblasts. We found that nhpESC 4706 expressed all six nAChR subunits (α1, α5, α7, β1, β2, and γ/ϵ) as well as β-actin (Figure 1). After differentiation into fibroblasts, nhpESC continue to express nicotinic receptors in the presence or absence of 100 nM nicotine (Figure 1). As control, we examined the expression of nAChR subunits in the rhesus brain, muscle, and primary cultures of rhesus lung fibroblasts in the presence or absence of 100 nM nicotine added to the culture medium. We found that the appropriate subunits were expressed (Figure 1) as previously described in human tissues [34].

### Expression of stem cell markers after in vitro differentiation

**Figure 2:**
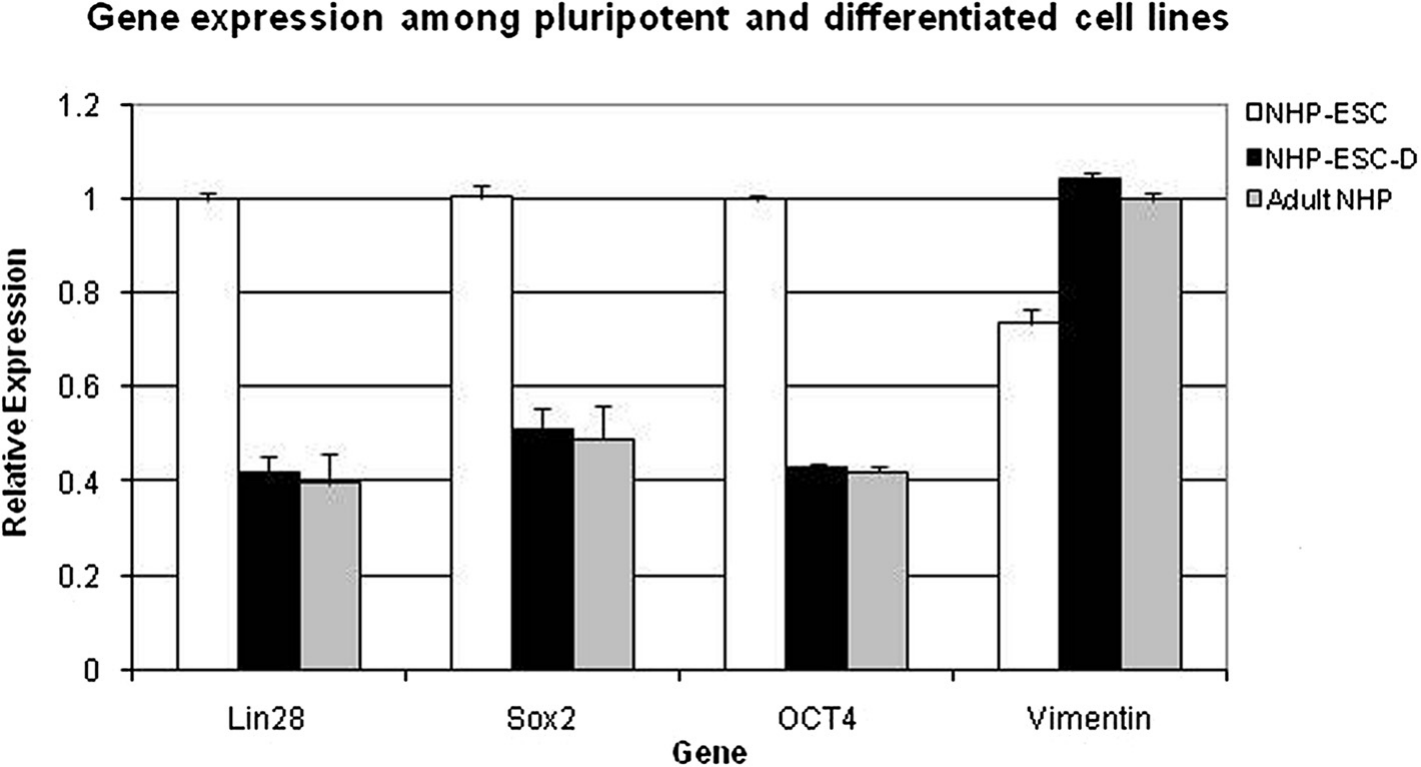
**Differentiated nhpESC expression profile is similar to primary lung fibroblasts.** After directed differentiation, gene expression patterns in NHP-ESC-D match the expression pattern of primate lung fibroblasts and are significantly different from pluripotent NHP-ESC, as determined by gene array analysis. We compared undifferentiated nhpESC, differentiated nhpESC, and primary NHP fibroblasts for expression of the pluripotency genes Lin28, Sox2, and OCT4, as well as the fibroblast gene vimentin. In all assays, gene expression of the differentiated nhpESC was similar to the primary NHP fibroblasts and not the pluripotent parental nhpESC line. Statistical tests used were ANOVA followed by Bonferroni’s post-test. Differences between NHP-ESC and NHP-ESC-D or adult NHP are significant (p < 0.01). NHP-ESC-D and adult NHP cells were not significantly different from one another for the expression of any genes.

**Figure 3:**
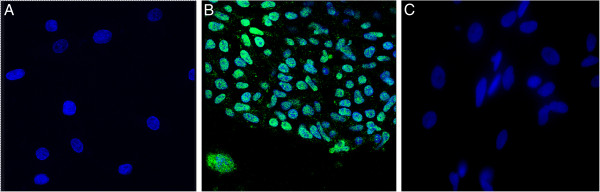
**Differentiated nhpESC do not express OCT4.** Cells were stained by immunofluorescence for the pluripotency marker OCT4. **A**: NHP primary lung fibroblasts served as negative controls for OCT4; **B**: undifferentiated nhpESC 4706 served as positive controls for OCT4; **C**: nhpESC 4706 differentiated into fibroblasts were negative for OCT4, similar to panel A primary lung fibroblasts.

**Figure 4:**
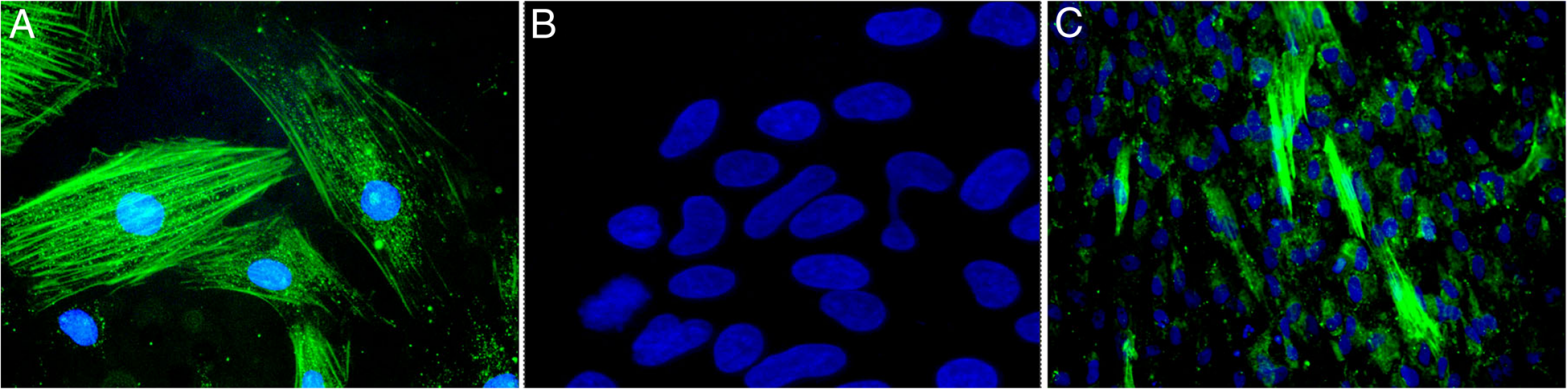
**Differentiated nhpESC express SMA.** Cells were stained by immunofluorescence for the fibroblast maker SMA. **A**: nhp primary lung fibroblasts served as positive controls for SMA; **B**: undifferentiated nhpESC 4706 served as negative controls for SMA; **C**: nhpESC 4706 differentiated into fibroblasts were positive for SMA, similar to panel A primary lung fibroblasts.

We found that *in vitro* differentiated fibroblasts had significantly decreased mRNA expression of the pluripotency markers Lin28, Sox2, and OCT4 as compared to undifferentiated controls (Figure 2), while the expression of the fibroblast differentiation marker vimentin increased significantly (Figure 2). We next confirmed these results using immunocytochemistry, and found OCT4 expression in undifferentiated nhpESC (Figure 3B), consistent with results by Navara et al. [12]. In addition, undifferentiated nhpESC were negative for expression of the fibroblast marker smooth muscle actin (SMA) (Figure 4B). In contrast, after directed differentiation of the nhpESC into fibroblasts, cells were negative for OCT4 (Figure 3C) and positive for SMA (Figure 4C), similar to our control adult rhesus lung fibroblast cultures, which were negative for OCT4 (Figure 3A) and positive for SMA (Figure 4A).

### Differentiated fibroblasts do not form teratomas

Although the parental undifferentiated nhpESC were able to form teratomas ([12]; personal communication), the hallmark of pluripotent cells, we confirmed that *in vitro* differentiated fibroblasts cannot form teratomas, using the same Core facility and methods. Cells were injected into SCID mice in 6 separate injections for a 0% success rate, comparing with an historical success rate of 92% for teratoma formation after injection of pluripotent stem cells in the Core facility [35]. Histological analysis of the injected testes four or more months after injection showed an apparent injection site with some inflammation, but no evidence of tumor formation (Additional file 1: Figure S1). Therefore, we conclude that these cells are differentiated and do not retain the ability to form teratomas.

### Fibroblast phenotype

**Figure 5:**
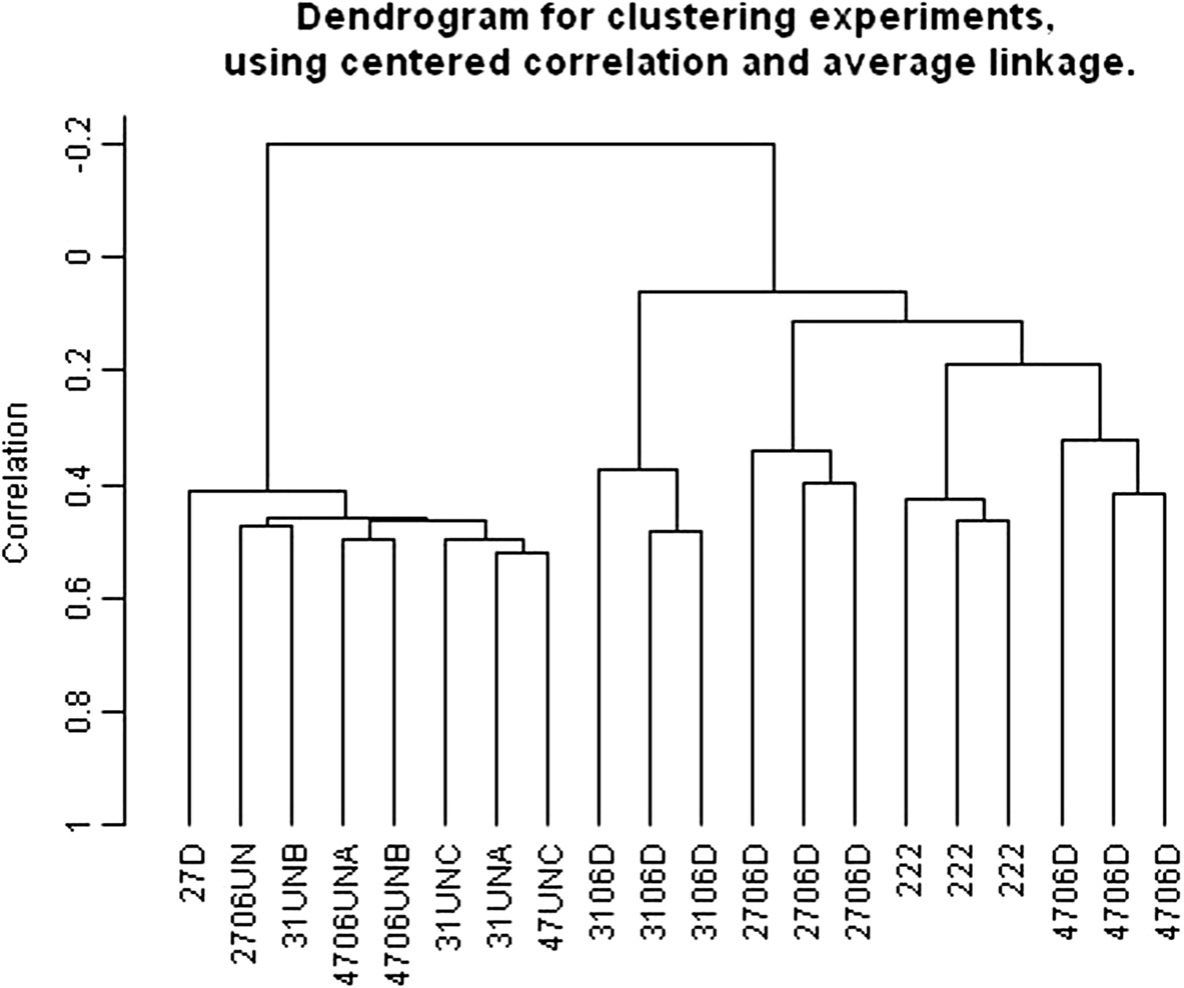
**Differentiated nhpESC are more similar to primary NHP fibroblasts than their parental cells with respect to global gene expression analysis.** Affymetrix gene array analysis was done to assay global gene expression patterns. The lines were then grouped according to similarities in gene expression using cluster analysis. This shows that *in vitro* differentiated fibroblast-like cells (4706D, 3106D, 2706D) are more similar to primary adult lung fibroblasts (222) than with the parental cells from which they were derived (4706UN, 2706UN, 3106UN).

We next compared global gene expression patterns using an Affymetrix non-human primate gene expression array. We compared primary NHP lung fibroblast cells with nhpESC before and after directed differentiation. We determined that the gene expression profiles of *in vitro* differentiated fibroblasts are very similar to cultured primary lung fibroblasts. In fact, we found that differentiated nhpESC have a global expression pattern that is closer to that of unrelated cultured lung fibroblasts than to the parental undifferentiated nhpESC (Figure 5).

**Figure 6:**
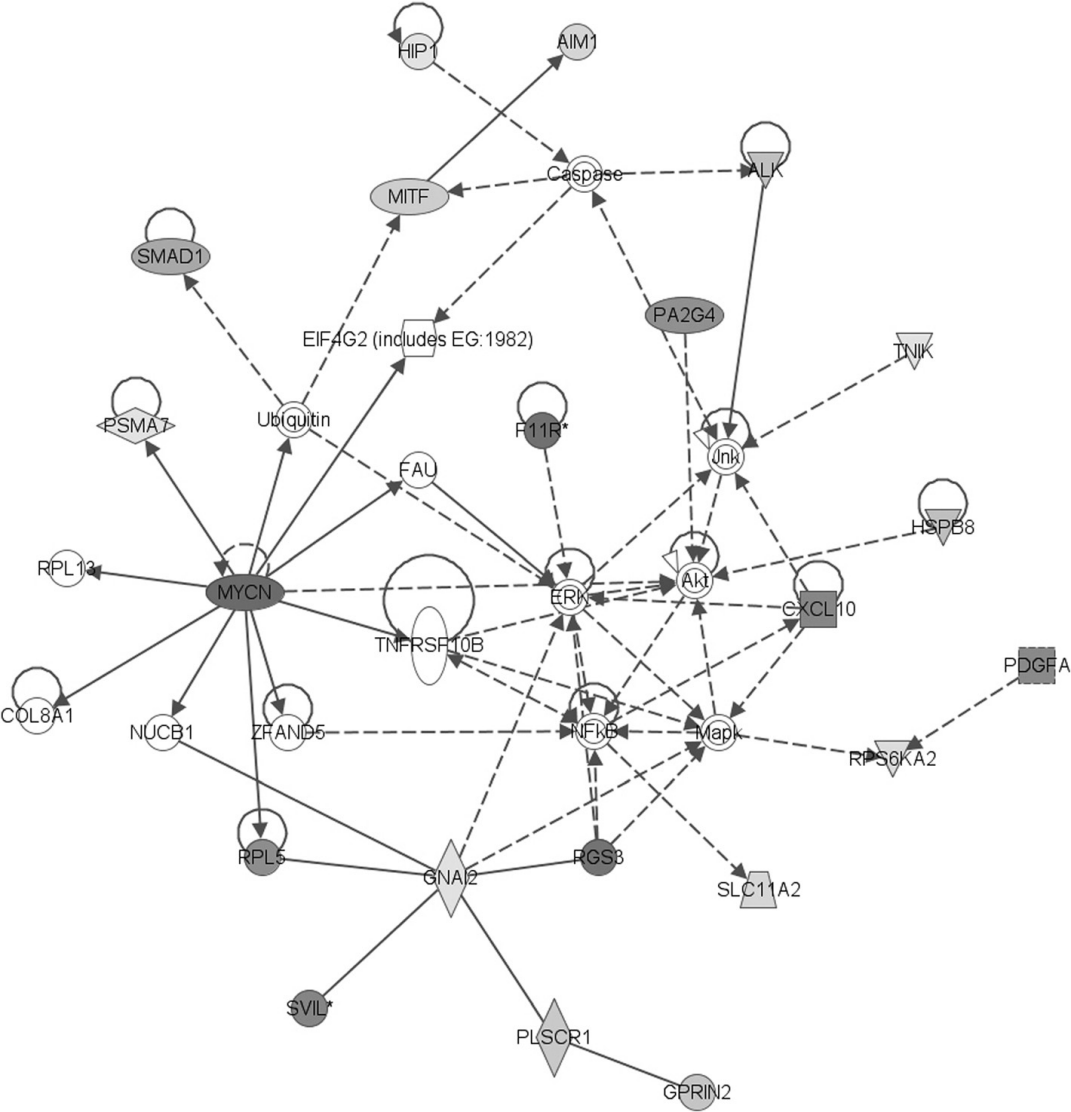
**Network profiling shows a central role for N-myc.** Using Ingenuity Analysis to compare the expression profiles of fibroblasts differentiated in the presence of nicotine versus controls, we find that the cell cycle signaling profile has a large number of genes which are altered (indicated by colored shading) as a result of nicotine exposure during differentiation.

Nicotine-exposed differentiated samples did not cluster separately from unexposed differentiated samples suggesting that the overall the gene expression profiles were similar. However, there were some significant differences in gene expression between these two groups. There is a finite group of less than 100 genes and gene loci that are different between nicotine exposed and unexposed cultures, P < 0.001. Using Ingenuity analysis, we found that the genes that are changed are not representative of all pathways, as would be expected by chance, and cell cycle-related genes are over-represented in the cohort. We found that expression of genes in cell cycle signaling pathways are generally decreased by nicotine exposure during differentiation, with the exception of SMAD, which showed increased in expression (Figure 6). These same genes are increased in adult fibroblasts that are exposed continuously to nicotine for 3 passages; thus, the decrease in cell cycle signaling pathway is specific to nicotine exposure during differentiation and is not a general effect of nicotine on cultured fibroblasts.

**Figure 7:**
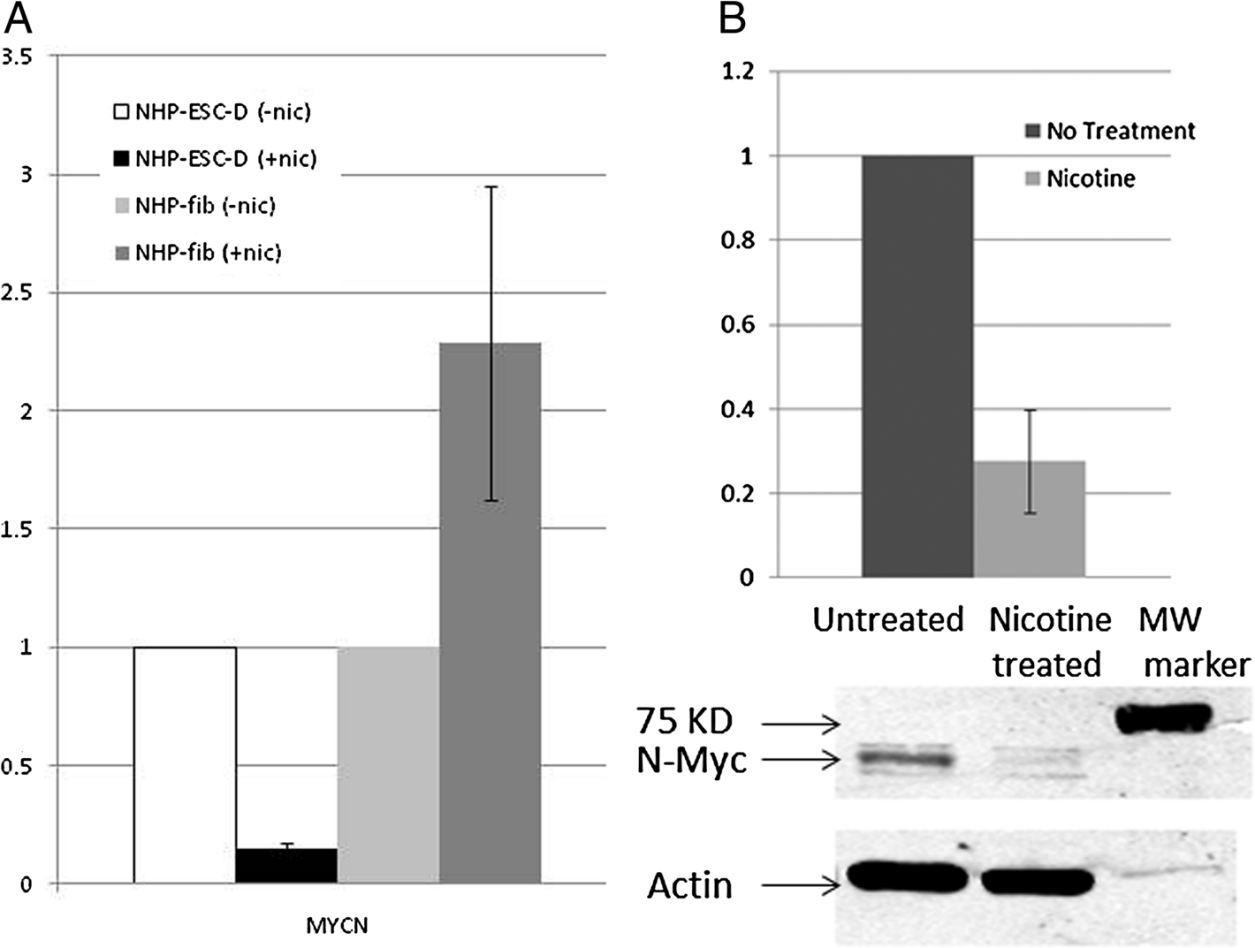
**N-myc is decreased after differentiation in the presence of nicotine. A**. The decrease in N-myc expression caused by nicotine exposure during differentiation was detected by array and confirmed here by real-time PCR. In addition, this demonstrates that fibroblasts exposed to nicotine after differentiation have the opposite response. **B**. N-myc protein expression was quantified by immunoblotting at passages 8, 9, and 13. The top panel shows decreased N-myc protein after nicotine treatment as determined by LiCor imaging analysis, with fluorescence intensity for each sample normalized to β-actin. The bottom panel is a representative blot, showing protein expression at passage 8. Changes between untreated and nicotine treated cells are significant, P < 0.05 for both qPCR and immunoblotting analyses.

We confirmed the expression pattern of N-myc. The qPCR data demonstrate a significant decrease in N-myc in nhpESC differentiated cells in the presence of nicotine compared to unexposed differentiated nhpESC in this comparison (Figure 7A). Due to the important role of N-myc in lung development and our interest in this gene, we also confirmed that N-myc protein levels were decreased in differentiated fibroblasts that were exposed to nicotine, as compared to untreated differentiated fibroblasts (Figure 7B).

#### Nicotine increases doubling time of differentiated nhpESC

**Figure 8:**
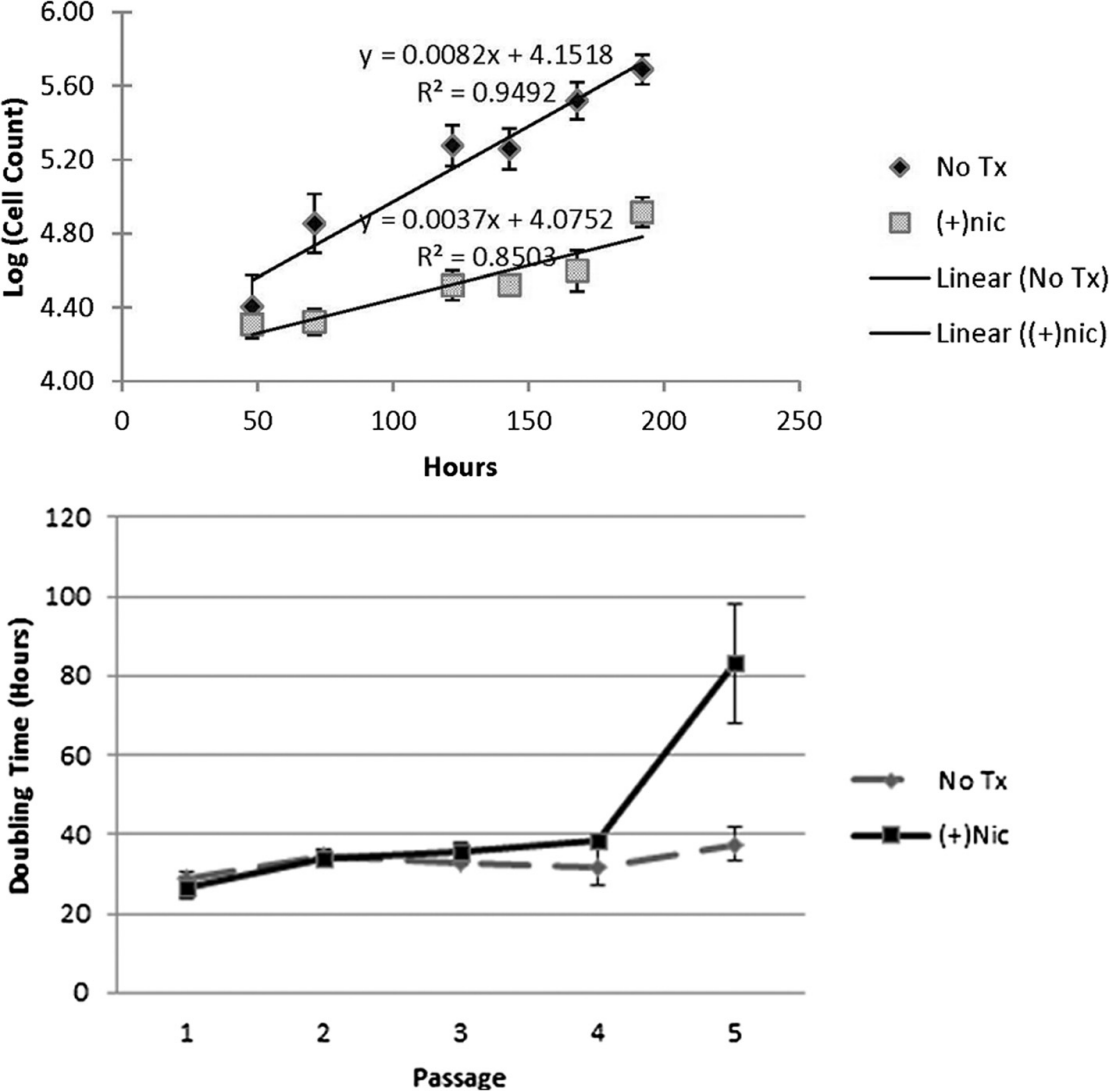
**Nicotine exposure during differentiation decreases cell counts and increases doubling time.** nhpESC were differentiated in the presence or absence of 100 nM nicotine. Doubling time was measured by cell counts through passage 5 after differentiation. Data is expressed as cell counts at passage five, with error bars demonstrating standard deviation, p < 0.001 (upper panel), and this data was converted into doubling time, show in hours (lower panel). Cells differentiated in the presence of nicotine had a higher doubling time, which was significantly slower than control starting at passage 5.

N-myc is known to promote proliferation through several mechanisms, including stimulating ribosome biogenesis and by inhibiting the cell cycle repressor p15Ink4b (reviewed by [41]. Since Ink4b is a negative regulator of the cell cycle, expression of N-myc allows cells to cycle more rapidly [42,43]. In addition to its effects on proliferation, N-myc has been demonstrated to have a role in the regulation of cell death, with overexpression of N-myc leading to an increase apoptosis (reviewed by [41]). In order to determine the downstream effect of decreased N-myc expression caused by nicotine during differentiation, we performed cell counts at each passage after differentiation through passage 5 and used regression analysis to analyze the counts. Using this data, we determined the doubling time of nhpESC after differentiation into fibroblasts in the presence or absence of nicotine starting at passage 1 after differentiation. We found that as nhpESC differentiated into fibroblasts in the presence of nicotine, the doubling time increased as compared to control cultures differentiated in the absence of nicotine. This difference developed over time as cells were passaged, and was significant at passage 5 (Figure 8).

## Discussion

The acetylcholine signaling pathway has been established as functional in mouse ESC (mESC), and publications have demonstrated that mESC express choline acetyltransferase and secrete acetylcholine into the culture medium [44-46]. There are currently a few studies that examine the effect of nicotine on ESC culture and differentiation; however, they have conflicting results. Nicotine increases the expression of OCT4 and Rex1 in undifferentiated mESC [47]. However, in a separate study, nicotine inhibited attachment of undifferentiated hESC to matrigel and led to a corresponding decrease in OCT4 staining, although the cells remained pluripotent [48]. Thus, the effect of nicotine on the expression of pluripotency markers in undifferentiated cells is unclear. Related studies have examined induced pluripotent stem cells (iPSC) from mice, and these studies found that nicotine increased undifferentiated stem cell proliferation [49,50].

In this study, we document for the first time the presence of nAChR on primate pluripotent stem cells. Since ESC differentiation can serve as a model for development, these results are a critical discovery, because they imply that with the presence of these receptors it is possible for maternal nicotine to affect the earliest stages of embryonic development, including the ability to differentiate into all the cell types in the body.

During active smoking, smokers have been shown to obtain a nicotine concentration of 100 nM in the serum [51]. In addition, smokers have a minimum serum-nicotine concentration that they maintain throughout the day, which has been shown to average 100 nM but ranges between 10 nM and 200 nM for light and heavy smokers, respectively [52]. Furthermore, since nicotine is known to cross the placenta, this dose may also be relevant to fetal exposure [1]. Our data show that nAChR are expressed on nhpESC, therefore, we tested the ability of a physiologically-relevent dose of nicotine, 100 nM, to affect differentiation. Using an *in vitro* differentiation method, we found that nhpESCs differentiated into fibroblasts in the presence of nicotine do not have any obvious differences in cell appearance (Figures 3 and 4); however, they show significant differences in gene expression patterns especially with respect to cell cycle-related genes, most notably N-myc. N-myc is decreased in expression in the differentiated fibroblasts. This effect is most dramatic in the early passages after differentiation (passages 1–4); and in some experiments remained significantly decreased through the final passage examined, passage 10. The decreased expression of N-myc is not mimicked by long-term exposure of adult lung fibroblasts to nicotine (Figure 7). These expression differences are unique to nhpESCs differentiated in the presence of nicotine, and are not changed in adult NHP fibroblasts passaged in nicotine for an equivalent period of time. This implies that they are disregulated during differentiation, and this disregulation is maintained multiple passages after differentiation.

The effects of nicotine exposure on adult fibroblasts has been studied previously [34,53] and many others have examined the effect of nicotine on bronchial epithelial cells and lung cancer cells [54-62]. In the lung, normal fibroblasts and epithelium express functional nAChR and these receptors are overexpressed in lung cancer [34,54]. Signaling through these receptors in the lung leads to activation of signaling pathways consistent with lung cancer, including the MAPKs and AKT [34,63]. In addition, short term experiments, including those up to 48 hours, indicated that bronchial epithelial cells increased expression of nAChR and have increased nicotinic signaling after exposure [56] and fibroblasts increase fibronectin and nAChR expression [53]. Thus, both *in vivo* and *in vitro* studies show that nAChR have an endogenous function in the lung, and that exposure to nicotine alters characteristics of the lung epithelium and the supporting fibroblasts. A murine lung explant model has also been used to examine nicotine toxicity, whereby embryonic lungs were isolated from normal mice halfway through gestation and then exposed to nicotine in culture [40]. However, the published data, whereby lung explants, which are composed of already differentiated (although immature) cells, are exposed to nicotine after they are placed in culture, are not likely to reflect the effects of nicotine on the differentiation process. Thus, none of these *in vivo* or *in vitro* studies can model the effect of nicotine on the differentiation process itself, since these cells are already differentiated at the time that they are exposed.

Several cell cycle genes were significantly different between nicotine exposed and unexposed groups in this study. The determination that N-myc was decreased in expression was particularly interesting due to its role in lung organogenesis. N-myc has been established as an essential regulator of lung organogenesis using murine developmental models [64-68]. Our data suggest that nicotine downregulates N-myc through intracellular signaling initiated by the binding of nicotine to the muscle-type and/or α7 neuronal nAchR. Our data also show that nhpESC cultures differentiated into fibroblasts in the presence of nicotine have a slower doubling time, indicative of either a slower proliferation rate or an increased cell death rate in the culture. This data, together with publications that have shown that N-myc is a mediator of both cell proliferation and cell death, suggest that as a result of nicotine inhibition of N-myc expression, fibroblasts proceed through the cell cycle at a significantly slower pace than do fibroblasts that are not exposed to nicotine during differentiation.

## Conclusions

In the lung, prenatal cigarette smoke exposure causes decreased lung function at birth and into childhood [69-74] as well as increased incidence of asthma and allergies [74]. The mechanisms by which cigarette smoke has these effects are not known; however, it is modeled in non-human primates exposed during pregnancy to nicotine. Studies find that although there are thousands of chemicals in cigarette smoke, NHP infants subjected to nicotine alone via a maternal exposure during development have increased thickness of the inner airway wall and altered complexity of branching, decreased respiratory volumes, and altered response to airway challenges [4,5,40,75]. The ability of nicotine to alter N-myc signaling in nhpESC may be one mechanism by which maternal cigarette smoking adversely affects embryonic lung development, leading to disease later in life.

## Abbreviations

FBS: Fetal bovine serum; FGF: Fibroblast growth factor; hESC: Human embryonic stem cell; NHP: Non-human primate; KO-DMEM: Knockout dulbeccos modified eagle’s medium; MEF: Mouse embryonic fibroblast; nAChR: Nicotinic acetylcholine receptor; nhpESC: nOn-human primate embryonic stem cell; PBS: Phosphate buffered saline; qPCR: Quantitative real-time polymerase chain reaction; RT-PCR: Reverse transcription polymerase chain reaction; Tx: Triton X100.

## Competing interests

Financial competing interests

In the past five years have you received reimbursements, fees, funding, or salary from an organization that may in any way gain or lose financially from the publication of this manuscript, either now or in the future? Is such an organization financing this manuscript (including the article-processing charge)? If so, please specify. **No**

Do you hold any stocks or shares in an organization that may in any way gain or lose financially from the publication of this manuscript, either now or in the future? If so, please specify. **No**

Do you hold or are you currently applying for any patents relating to the content of the manuscript? Have you received reimbursements, fees, funding, or salary from an organization that holds or has applied for patents relating to the content of the manuscript? If so, please specify. **No**

Do you have any other financial competing interests? If so, please specify. **No**

Non-financial competing interests

Are there any non-financial competing interests (political, personal, religious, ideological, academic, intellectual, commercial or any other) to declare in relation to this manuscript? If so, please specify. **No**

## Authors’ contributions

DLC conceived of the project, obtained funding, designed experiments, performed differentiation experiments and data analysis, and wrote the manuscript. ABY, BMC, and LMW assisted with research design, performed experiments and data analysis, and assisted with writing the manuscript. TNK, JB, and VE assisted with research design and performed experiments. CAC performed pathology analysis of teratomas. All authors read and approved the final manuscript.

## Supplementary Material

Additional file 1 **Differentiated nhpESC do not form Teratomas.** Left Panel: Histology of testes from nude mice 6 months after injection with approximately 5x105 nhpESC. Histology is the same as Right Panel: negative control, wherein testes were injected with adult primary NHP fibroblasts. Table of genes significantly changed after analysis of GeneChip Rhesus Macaqua Genome Array from Affymetrix. The table lists genes and genetic loci that were changed as a result of 100 nM nicotine treatment during differentiation of nhpESC from pluripotency to fibroblast-like cells, p<0.001. Click here for file
