# Supplementary material for: Nicotine exposure during differentiation causes inhibition of N-myc expression
Source: Respir Res. 2013 Nov 5;14(1):119. doi: 10.1186/1465-9921-14-119 (PMC3828478; doi:10.1186/1465-9921-14-119)
Supplement: Additional file 1 — Differentiated nhpESC do not form Teratomas. Left Panel: Histology of testes from nude mice 6 months after injection with approximately 5x10^5^ nhpESC. Histology is the same as Right Panel: negative control, wherein testes were injected with adult primary NHP fibroblasts. Table of genes significantly changed after analysis of GeneChip Rhesus Macaqua Genome Array from Affymetrix. The table lists genes and genetic loci that were changed as a result of 100 nM nicotine treatment during differentiation of nhpESC from pluripotency to fibroblast-like cells, p<0.001. [file 1465-9921-14-119-S1.pdf]

Supplementary Data

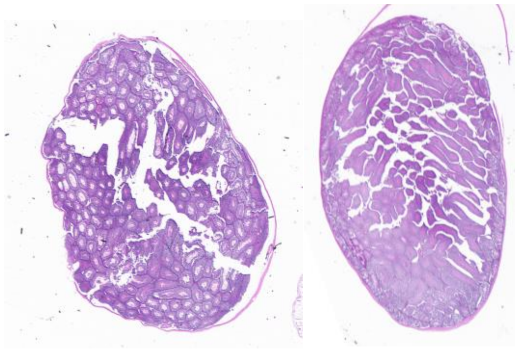

Supplementary Figure: Differentiated nhpESC do not form Teratomas. Left Panel: Histology of testes from nude mice 6 months after injection with approximately $5x10^5$ nhpESC. Histology is the same as Right Panel: negative control, wherein testes were injected with adult primary NHP fibroblasts.

Supplementary Table 1: Table of genes significantly changed after analysis of GeneChip Rhesus Macaqua Genome Array from Affymetrix. The table lists genes and genetic loci that were changed as a result of 100 nM nicotine treatment during differentiation of nhpESC from pluripotency to fibroblast-like cells, p<0.001.

	**Parametric p-value**	**Gene symbol**	**Description**
1	1.06E-05	LOC702700	similar to choline/ethanolaminephosphotransferase
2	1.67E-05	MYCN	v-myc myelocytomatosis viral related oncogene, neuroblastoma derived (avian)
3	2.82E-05	GSTT2	glutathione S-transferase theta 2
4	3.79E-05	SAMHD1	SAM domain and HD domain 1
5	6.12E-05	LOC705398	similar to ribosome associated membrane protein 4
6	7.01E-05	LOC696076	similar to cytochrome P450, family 2, subfamily U, polypeptide 1
7	7.96E-05	LOC708713	similar to F56A8.1
8	9.13E-05	LOC716117	similar to hairy/enhancer-of-split related with YRPW motif-like
9	9.25E-05	F11R	F11 receptor
10	9.84E-05	LOC697751	hypothetical protein LOC697751

11	0.000113	LOC704106	similar to guanine nucleotide binding protein (G protein), alpha activating activity polypeptide, olfactory type isoform 1
12	0.000113	LOC704953	similar to longevity assurance homolog 6
13	0.000116	CXCL10	chemokine (C-X-C motif) ligand 10
14	0.000129	SLC16A2	solute carrier family 16, member 2
15	0.000135	LOC706073	similar to cyclic nucleotide gated channel beta 1
16	0.000136	LOC719634	similar to regulator of G-protein signalling 3 isoform 5
17	0.000137	LOC711079	hypothetical protein LOC711079
18	0.000147	GPR158	G protein-coupled receptor 158
19	0.000153	LOC705497	similar to transforming, acidic coiled-coil containing protein 2 isoform d
20	0.000161	LOC721695	similar to Nucleoside diphosphate-linked moiety X motif 8, mitochondrial precursor (Nudix motif 8)
21	0.000162	LOC712589	similar to ATPase, Class VI, type 11A isoform b
22	0.000163	IFNA14	interferon, alpha 14
23	0.000165	LOC700632	similar to tumor protein p53 inducible nuclear protein 1
24	0.000165	LOC721873	similar to squamous cell carcinoma antigen recognized by T cells 1
25	0.000166	LOC707383	similar to amyloid beta (A4) precursor protein-binding, family B, member 1 interacting protein
26	0.000171	LOC693274	similar to NGFI-A binding protein 1
27	0.000197	LOC715297	similar to N-ethylmaleimide-sensitive factor
28	0.000203	LOC700715	similar to copine family member IX
29	0.000204	LOC701602	similar to inter-alpha (globulin) inhibitor H5
30	0.000209	LOC712994	similar to secreted modular calcium-binding protein 1 isoform 2
31	0.000221	LOC712932	similar to Ku70-binding protein 3
32	0.000228	FMO4	flavin containing monooxygenase 4
33	0.000243	LOC715297	similar to N-ethylmaleimide-sensitive factor
34	0.00025	MT	mitochondrial malonyltransferase
35	0.000254	SRD5A2	steroid-5-alpha-reductase, alpha polypeptide 2 (3-oxo-5 alpha-steroid delta 4-dehydrogenase alpha 2)
36	0.00026	LOC712374	similar to CG14535-PA
37	0.000265	LOC713280	similar to meningioma 1
38	0.000285	LOC695267	similar to supervillin isoform 2
39	0.000287	LOC695267	similar to supervillin isoform 2
40	0.000292	CXCL10	chemokine (C-X-C motif) ligand 10
41	0.000298	LOC702886	similar to E74-like factor 4 (ets domain transcription factor)

42	0.000304	LOC719482	similar to chromosome 10 open reading frame 64
43	0.000304	LOC705975	similar to cyclin-dependent kinase-like 1
44	0.000316	LOC700866	hypothetical protein LOC700866
45	0.00032	LOC707725	similar to platelet-derived growth factor alpha isoform 2 preproprotein
46	0.000336	LOC697003	similar to ovary-specific acidic protein
47	0.000341	F11R	F11 receptor
48	0.000355	LOC705032	similar to SLIT and NTRK-like family, member 5
49	0.000365	PA2G4	proliferation-associated 2G4, 38kDa
50	0.000372	LOC704106	similar to guanine nucleotide binding protein (G protein), alpha activating activity polypeptide, olfactory type isoform 1
51	0.000373	LOC706437	hypothetical protein LOC706437
52	0.000401	LOC715100	similar to mitochondrial ribosomal protein L4 isoform a
53	0.000403	LOC700135	similar to Probable ubiquitin carboxyl-terminal hydrolase FAF-X (Ubiquitin thioesterase FAF-X) (Ubiquitin-specific-processing protease FAF-X) (Deubiquitinating enzyme FAF-X) (Fat facets protein-related, X-linked) (Ubiquitin-specific protease 9, X c...
54	0.000404	LOC706304	hypothetical protein LOC706304
55	0.000406	CPA4	carboxypeptidase A4
56	0.000506	SMAD1	SMAD family member 1
57	0.000516	LOC699343	similar to spermatogenesis associated 11
58	0.000568	LOC708498	similar to hippocampus abundant transcript-like 1
59	0.000588	LOC709833	hypothetical protein LOC709833
60	0.000593	LOC715058	similar to zinc finger, matrin type 5
61	0.000605	LOC694299	similar to serum deprivation response protein
62	0.000611	IVD	isovaleryl Coenzyme A dehydrogenase
63	0.000613	LOC715394	similar to laminin alpha 2 subunit precursor
64	0.000616	LOC696973	similar to SWI/SNF-related matrix-associated actin-dependent regulator of chromatin a2 isoform a
65	0.000633	LOC716726	similar to isocitrate dehydrogenase 3, beta subunit isoform b precursor
66	0.000638	HSPB8	heat shock 22kDa protein 8
67	0.000646	ALK	anaplastic lymphoma kinase (Ki-1)
68	0.000649	ZNF436	zinc finger protein 436
69	0.000654	LOC713549	similar to 3-hydroxysteroid epimerase
70	0.00067	LOC694369	similar to G protein-regulated inducer of neurite outgrowth 2 (GRIN2)
71	0.000673	LOC694818	similar to cytochrome b reductase 1

72	0.000674	OGFR	opioid growth factor receptor
73	0.000676	LOC695466	similar to scavenger receptor class F, member 2 isoform 2
74	0.000697	LOC713232	similar to phospholipid scramblase 1
75	0.000704	LOC693823	similar to LPS-responsive vesicle trafficking, beach and anchor containing
76	0.000718		
77	0.000735	LOC693823	similar to LPS-responsive vesicle trafficking, beach and anchor containing
78	0.000749		Cytidine monophosphate N-acetylneuraminic acid synthetase
79	0.000753	LOC715394	similar to laminin alpha 2 subunit precursor
80	0.000761	LOC696973	similar to SWI/SNF-related matrix-associated actin-dependent regulator of chromatin a2 isoform a
81	0.000763	SLC11A2	solute carrier family 11 (proton-coupled divalent metal ion transporters), member 2
82	0.000764	LOC697117	similar to absent in melanoma 1
83	0.000764	LOC695267	similar to supervillin isoform 2
84	0.000792	LOC702799	hypothetical protein LOC702799
85	0.000796		Similar to 60S ribosomal protein L21
86	0.000823	LOC709054 /// LOC711640	hypothetical protein LOC709054 /// similar to CDK2-associated protein 2
87	0.000831	GNAI2	guanine nucleotide binding protein (G protein), alpha inhibiting activity polypeptide 2
88	0.000845	LOC715853	similar to interphase cytoplasmic foci protein 45
89	0.000866	SEMG1	semenogelin I
90	0.000891	LOC715195	similar to SRR1-like protein
91	0.000897	TNIK	TRAF2 and NCK interacting kinase
92	0.000911	LOC714530	similar to growth arrest-specific 2 like 1 isoform a
93	0.000918	LOC720175	similar to proteasome (prosome, macropain) subunit, alpha type 7
94	0.00093	LOC721230	similar to tumor suppressing subtransferable candidate 5
95	0.000935	LOC722609	similar to WD repeat domain 34
96	0.000952	LOC716166	similar to Sjogrens syndrome/scleroderma autoantigen 1
97	0.000987	LOC701345	similar to neurogenin 2
98	0.000989	LOC722330	similar to phosphofructokinase, platelet
99	0.000996	LOC706437	hypothetical protein LOC706437
